# Third International Consensus Conference on lesions of uncertain malignant potential in the breast (B3 lesions)

**DOI:** 10.1007/s00428-023-03566-x

**Published:** 2023-06-17

**Authors:** Constanze Elfgen, Cornelia Leo, Rahel A. Kubik-Huch, Simone Muenst, Noemi Schmidt, Cecily Quinn, Sorcha McNally, Paul J. van Diest, Ritse M Mann, Zsuzsanna Bago-Horvath, Maria Bernathova, Peter Regitnig, Michael Fuchsjäger, Daniela Schwegler-Guggemos, Martina Maranta, Sabine Zehbe, Christoph Tausch, Uwe Güth, Eva Maria Fallenberg, Simone Schrading, Ashutosh Kothari, Martin Sonnenschein, Gert Kampmann, Janina Kulka, Jean-Christophe Tille, Meike Körner, Thomas Decker, Sigurd F. Lax, Martin Daniaux, Vesna Bjelic-Radisic, Stephanie Kacerovsky-Strobl, Rosaria Condorelli, Michael Gnant, Zsuzsanna Varga

**Affiliations:** 1 Breast-Center Zurich, Zurich, Switzerland; 2 University of Witten-Herdecke, Witten, Germany; 3 Breast Center, Kantonsspital Baden, Baden, Switzerland; 4 Department of Radiology, Kantonsspital Baden, Baden, Switzerland; 5 Institute of Medical Genetics and Pathology, University Hospital Basel, Basel, Switzerland; 6 Department of Radiology, University Hospital Basel, Basel, Switzerland; 7 Irish National Breast Screening Program & Department of Histopathology, St. Vincent’s University Hospital Dublin and School of Medicine, University College Dublin, Dublin, Ireland; 8 Radiology Department, St. Vincent University Hospital, Dublin, Ireland; 9 Department of Pathology, University Medical Center Utrecht, Utrecht, The Netherlands; 10 Department of Radiology, Radboud University Medical Center, Nijmegen, The Netherlands; 11 Department of Radiology, The Netherlands Cancer Institute, Amsterdam, The Netherlands; 12 Department of Pathology, Medical University Vienna, Vienna, Austria; 13 Department of Radiology and Nuclear Medicine, Medical University Vienna, Vienna, Austria; 14 Diagnostic and Research Institute of Pathology, Medical University Graz, Graz, Austria; 15 Division of General Radiology, Department of Radiology, Medical University Graz, Graz, Austria; 16 Department of Radiology, County Hospital Aarau, Aarau, Switzerland; 17 Department of Gynecology, County Hospital Chur, Chur, Switzerland; 18 Radiology Section, Breast Center Stephanshorn, St. Gallen, Switzerland; 19 Department of Diagnostic and Interventional Radiology, School of Medicine & Klinikum Rechts der Isar, Technical University of Munich (TUM), Munich, Germany; 20 Department of Radiology, County Hospital Lucerne, Lucerne, Switzerland; 21 Breast Surgery Unit, Guy’s and St Thomas’s NHS Foundation Trust, London, UK; 22 Radiology Section, Breast Center Lindenhofgruppe AG, Bern, Switzerland; 23 Centro di Radiologia e Senologia Luganese, Lugano, Switzerland; 24 Department of Pathology, Forensic and Insurance Medicine, Semmelweis University Budapest, Budapest, Hungary; 25 Division of Clinical Pathology, University Hospital Geneva, Geneva, Switzerland; 26 Pathologie Länggasse, Ittigen, Switzerland; 27 Breast Pathology, Reference Centers Mammography Münster, University Hospital Münster, Münster, Germany; 28 Department of Pathology, Hospital Graz II, Graz, and School of Medicine, Johannes Kepler University Linz, Linz, Austria; 29 BrustGesundheitZentrum Tirol, University Hospital Innsbruck, Innsbruck, Austria; 30 Breast Unit, Helios University Hospital, University Witten/Herdecke, Witten, Germany; 31 Breast Center, Franziskus Hospital, Vienna, Austria; 32 IOSI, Ente Ospedaliero Cantonale, Bellinzona, Switzerland; 33 Comprehensive Cancer Center, Medical University Vienna, Vienna, Austria; 34 Department of Pathology and Molecular Pathology, University Hospital Zürich, Zürich, Switzerland

**Keywords:** Vacuum-assisted biopsy, B3 lesion, Uncertain malignant potential, ADH, LN, FEA, Radial Scar, Phyllodes tumor, Papilloma, Core-needle biopsy, Consensus, Vacuum-assisted excision, Breast surgery

## Abstract

The heterogeneous group of B3 lesions in the breast harbors lesions with different malignant potential and progression risk. As several studies about B3 lesions have been published since the last Consensus in 2018, the 3rd International Consensus Conference discussed the six most relevant B3 lesions (atypical ductal hyperplasia (ADH), flat epithelial atypia (FEA), classical lobular neoplasia (LN), radial scar (RS), papillary lesions (PL) without atypia, and phyllodes tumors (PT)) and made recommendations for diagnostic and therapeutic approaches. Following a presentation of current data of each B3 lesion, the international and interdisciplinary panel of 33 specialists and key opinion leaders voted on the recommendations for further management after core-needle biopsy (CNB) and vacuum-assisted biopsy (VAB). In case of B3 lesion diagnosis on CNB, OE was recommended in ADH and PT, whereas in the other B3 lesions, vacuum-assisted excision was considered an equivalent alternative to OE. In ADH, most panelists (76%) recommended an open excision (OE) after diagnosis on VAB, whereas observation after a complete VAB-removal on imaging was accepted by 34%. In LN, the majority of the panel (90%) preferred observation following complete VAB-removal. Results were similar in RS (82%), PL (100%), and FEA (100%). In benign PT, a slim majority (55%) also recommended an observation after a complete VAB-removal. VAB with subsequent active surveillance can replace an open surgical intervention for most B3 lesions (RS, FEA, PL, PT, and LN). Compared to previous recommendations, there is an increasing trend to a de-escalating strategy in classical LN. Due to the higher risk of upgrade into malignancy, OE remains the preferred approach after the diagnosis of ADH.

## Introduction

The worldwide most commonly used pathologic classification for breast lesions is the B-classification. The B3 lesions represent a heterogeneous group of diseases characterized by several overlapping findings in imaging and histologically distinct and defined entities. Some of these entities are considered biologically of uncertain malignant potential and progression risk; others cannot be diagnosed with certainty on a biopsy [1–5]. Therefore, the diagnostic procedures are paramount for the appropriate clinical management [1, 2]. Over the past two decades, various adjustments to the management approach have been made based on newly available data on disease-specific survival and biological upgrade in subsequent surgical specimens [1, 2, 6]. In order to avoid overtreatment and with a perspective of de-escalation, several adjustments in therapeutic recommendations towards have been made. More than 40,000 VAB-based diagnoses have been reported in the Swiss Minimal-Invasive Breast Biopsy (MIBB) Working Group database, of which 17% representing B3 lesions. Most of these lesions were found on mammography (65%), or ultrasound (29%), while only a small subset (6%) was seen on MRI [2]. Different therapeutic options, i.e., active surveillance, VAB, and OE, are used for B3 lesions, and suggestions are continually reassessed in the light of new scientific data. At the 3rd International Consensus Conference on B3 lesions, the six most relevant B3 lesions — atypical ductal hyperplasia (ADH), flat epithelial atypia (FEA), classical lobular neoplasia (LN), radial scar (RS), papillary lesions (PL) without atypia, and phyllodes tumors (PT) of benign and borderline categories — were discussed along with suggestions for diagnostic and therapeutic approaches. Innsbruck hosted the Consensus Conference as part of the joined Congress of the Swiss and Austrian Senology Societies in 2022.

## Methodology

The objective of this conference was defined as a review and discussion of the evidence levels and subsequent management recommendations based on an expert consensus that have emerged since the 2nd Consensus Conference on B3 lesions, which was held in Zurich, Switzerland in 2018. In addition, a systematic literature review as well as timely modified recommendations from other national and international guidelines such as the UK NHS breast screening multidisciplinary working group guidelines, the AGO Breast Committee of the German Society of Gynecology and Obstetrics, the European Society of Breast Imaging, and S3 guidelines of the German Cancer League were included in the discussion at this conference [1, 3, 7–9]. Similar to the recommendations from the 2nd Consensus Conference, the debate included a proposed upper risk limit of 5% upgrade for invasive carcinoma and 10% upgrade for ductal carcinoma in situ (DCIS) in order to refer to radiologic surveillance [2]. Surgeons, gynecologists, medical oncologists, radiologists, and pathologists made up the majority of panelists and attendees at the Joint Congress of the Swiss and Austrian Senology Societies 2022. The MIBB working group chose the panel, which was made up of a sizable interdisciplinary group of 11 pathologists, 12 radiologists, and 10 specialized gynecologists/specialized medical oncologists/breast surgeons from seven European countries. All panel members are renowned key opinion leaders in their field and have been actively participating in research on B3 lesions.

In addition, more than 100 participants of the consensus conference were invited to vote without being considered for the analysis of the Consensus Conference recommendations. The panelists and conference attendees voted separately on each question after a team of pathologists and radiologists presented the particular B3 lesion with focused review of the literature between 2018 and 2022. The MIBB data were not used to guide the discussion. The panel at this conference made a distinction between diagnostic core biopsy (CNB), a diagnostic or therapeutic vacuum assisted biopsy (VAB), or, alternatively, a secondary therapeutic vacuum-assisted excision (VAE), open surgical excision (OE), or no further intervention with follow-up only.

For each of the six B3 lesions panelists and participants were asked to answer the following three voting questions:

1. If a core-needle biopsy (CNB) returned as B3 lesion on histology, should the lesion be excised?
2. If so, should it be excised using vacuum-assisted biopsy (VAB) or open surgical excision (OE)?
3. If the VAB returned a B3 lesion on histology and if the lesion was completely removed on imaging, is surveillance acceptable or should a repeat VAB or OE be performed?

Following the voting, a panel discussion was held during which decisions regarding surveillance and consensus recommendations for the management of each B3 lesion were reached.

## Results

### Description of the most common B3 lesions

In the following paragraphs, the histological and radiological characteristics of the lesions are presented followed by the current evidence level of biological behavior and upgrade rate as well as the voting results of the Consensus Conference.

### Atypical ductal hyperplasia (ADH)

#### Histological features

ADH is a small low grade clonal intraductal lesion, either 2 mm in maximum diameter or involving only parts of a terminal ductulo-lobular unit (TDLU). It is a clonal proliferation and very often associated with calcifications [2, 4]. Since ADH is almost always completely negative for basal cytokeratins (e.g., CK5, CK5/6, CK14) and 100% (clonally) positive for estrogen receptors (ER), ancillary immunohistochemical tests are helpful in differentiating ADH from ductal hyperplasia of the usual type [2, 6]. ADH shares cytological and architectural similarities with low-grade DCIS but with partial involvement of TDLUs and/or uniform involvement to a limited extend. The distinction between ADH with uniform TDLU involvement is based on size/extent criteria. According to WHO classification, thresholds of 2 involved duct spaces with < 2 involved spaces [10] or size ≤ 2 mm in contiguous [11] are arbitrary. Because the extent of this neoplasia cannot be optimally assessed in minimally invasive biopsy specimens, the diagnosis of ADH cannot be made confidently from these specimens alone and low-grade DCIS cannot be excluded. Therefore, the European Working Group on Breast Screening Pathology proposed that such CNB results be referred to as “atypical ductal-type intraepithelial proliferation” (AEPD) instead of ADH, and the UK National Coordinating Committee for Breast Screening Pathology published this in 2001 [12]. Meanwhile, the European Guidelines for Mammographic Screening and their Pathology Supplement, as well as the most recent UK guidelines, mandate the use of the term “atypical ductal intraepithelial proliferation” (AEPD resp. AIDEP) in this situation [13–15]. The term AEPD/ AIDEP is therefore a terminus technicus used exclusively for the diagnosis on CNB or VAB. It is used to characterize a combination of findings in which the differential diagnosis ADH vs. DCIS is impossible. Therefore, the term AEPD/AIDEP is not included in the WHO tumor classification. However, the Editorial Committee for the Classification of Breast Tumors emphasizes that the ADH size criteria are based solely on findings on excisional biopsies and are therefore intended to serve only as “general guidelines.” The committee recommends that for core needle biopsies where the entire lesion may not be visible. These criteria can be used “conservatively” and with caution [4, 16]. Several histopathological factors have been researched with regards of their prognostic power for upgrading (including multifocality, the lack or presence of associated calcification, the presence of associated necrosis, number of cross sections >3, or the diagnostic biopsy method). However, no trustworthy histopathological feature that can reliably foretell upgrading in a following OE has been discovered to date [6, 17, 18]. The small size of ADH renders molecular studies difficult. The upgrade rate after “focal” ADH (lower limit of ADH) versus “full” ADH (upper limit of ADH) does not differ (10% vs. 11%) [19].

#### Radiological features

ADH is the most prevalent (28.4%) B3 lesion in the MIBB database, which might be related to the high number of mammography-guided VAB in the database. A clear majority (81.6%) of B3 lesions found as calcifications on mammography and, sometimes on ultrasound, is ADH [6]. Another study discovered a similar high incidence of ADH in B3 lesions (35%); but only 34% of ADH cases had accompanying calcifications. The majority of ADH lesions in this study were discovered within a mass lesion or in an architectural distortion, most likely since the study included a high percentage of CNB (61%), as well as seems to have some selection bias [14]. In a current systematic review, the pooled upgrade rate was 42% for ultrasound-guided CNB versus 23% for stereotactic VAB and 32% for MRI-guided VAB [20]. Under consideration of the different design of the reviewed studies, a significant correlation between guidance technique and needle caliber could be found [20]. ADH calcifications are frequently fine, pleomorphic, linear, or in segmental distribution, but various other imaging patterns can also occur [16]. The lesion exhibits non-specific characteristics on MRI, such as a focal area of non-mass enhancement and/or a small rounded to irregular mass [21].

#### Current evidence for underestimation after CNB/VAB

Since the last consensus conference, several studies reported an upgrade rate of ADH into malignancy (DCIS or invasive cancer) between 7.3 and 57% in targeted OE. Consequently, the majority of these studies recommended OE, although increasingly considering other options such as imaging follow-up after VAB, when the calcifications in clinical imaging have been completely removed [6, 19, 22–31]. In the MIBB database, patients with ADH underwent OE in the majority of cases (62.7%), and an upgrade rate of 25.2% was observed. Only 5% of upgrades revealed invasive carcinoma; the majority revealed DCIS [2]. Other studies demonstrated the substantial influence of biopsy technique and tissue amount (CNB vs. VAB), with the highest upgrade rate after CNB (31–78%) and a substantially lower rate after VAB (19–41%). This was especially evident in biopsies utilizing larger volumes with 7-8 Gauge (G) needles with a reduced upgrade rate no more than 19% [2, 16]. According to the most recent and largest meta-analysis, the rate of invasive carcinoma following OE was higher (29%) [20]. Risk factors for an upgrade to malignancy that can be thought of as favoring OE include [1] a lower amount of biopsy tissue (especially CNB or VAB with >8 G needles), [2] the lack of concordance between pathology results and imaging, [3] no correlation between calcifications in ADH and imaging, [4] residual calcifications after VAB, [5] the imaging size of the lesion (> 15mm), [6] the patient’s age (>50 years), and [7] multifocality of ADH in the biopsy specimens [6, 16–18]. However, it should be kept in mind that the complete removal of calcifications on clinical imaging does not rule out residual disease, which could be present without associated calcifications [6, 32]. The UK guidelines advise employing the so-called vacuum assisted excision (VAE), which uses larger VAB needles, and removing more specimen (at least 12 × 7-8 G, equivalent 4g of tissue) as an alternative to OE [7]. Additionally, it is known that ADH increases the risk of developing invasive ipsi- or contralateral breast cancer by up to 30% over the course of 25 years, necessitating yearly mammograms [33].

**Fig. 1:**
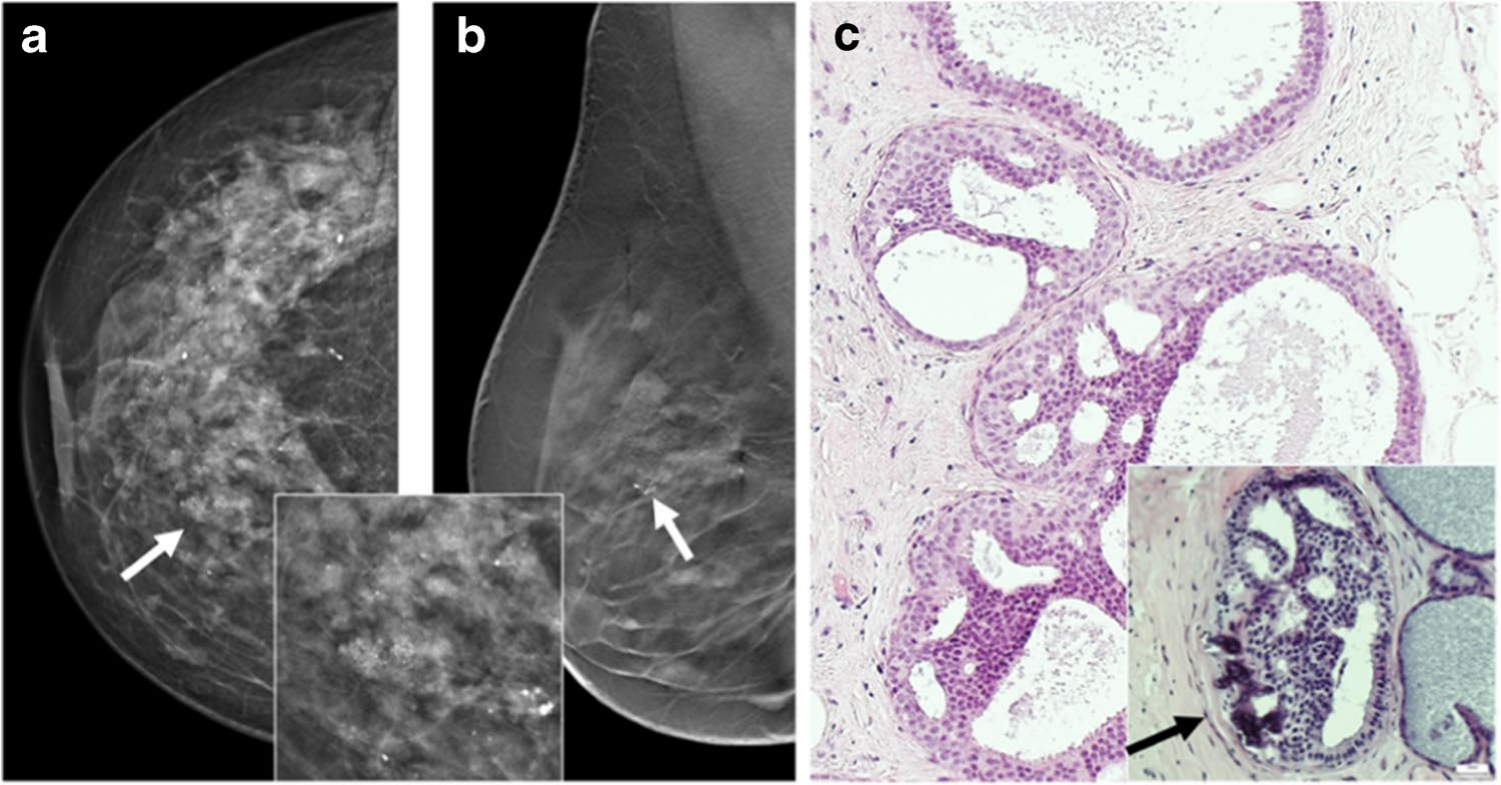
Atypical ductal hyperplasia (ADH). **a** and **b** Radiological presentation (**a** cranio-caudal (cc) view; **b** medio-lateral (mlo) tomosynthesis) of ADH with clustered microcalcifications seen on mammography (arrows). Inset shows higher magnification of clustered amorphous calcification which proved to be due to ADH. **c** Histological pictures show monotonous intraductal proliferation filling the whole cross section of the ductulus, building rigid lumina, and displaying an association to calcification as the histological correlation to the mammographically detected calcification seen in **a** and **b**. Inset shows abundant associated calcifications (H&E stain). H&E images: courtesy of Prof. Gad Singer, Pathology Kantonsspital Baden, Switzerland

Illustrative radiologic and morphological examples of ADH are shown in Fig. 1.

#### Consensus recommendation of the panel

The majority of the panelists (76%) recommended OE after ADH diagnosis on CNB, and more than half (58%) recommended OE after ADH diagnosis on diagnostic VAB. If the target lesion was entirely excised, observation and mammographic follow-up following diagnostic VAB was supported by 34% of the panelists, while a second therapeutic VAE was favored by 8% of the panelists (Tables 1 and 2). The use of larger VAB needles (7 or 8 G) and more extensive tissue sampling was supported to reduce the upgrade risk in ADH.

**Table 1:** Voting results of the panel

B3 lesion	If a CNB returned a B3 lesion on histology, should the lesion be excised?		If so, should it be excised using VAB or open OE?			If the VAB returned a B3 lesion on histology and if the lesion was completely removed on imaging, is surveillance acceptable or should a repeat VAB or OE be performed?		
	Yes	No	VAB	OE	No intervention	VAB	OE	No intervention
ADH	100%	0%	24%	76%	0%	8%	58%	34%
FEA	92%	8%	60%	38%	2%	0%	0%	100%
LN	86%	14%	78%	22%	0%	5%	5%	90%
PL	92%	8%	55%	45%	0%	0%	0%	100%
PT	92%	8%	6%	94%	0%	0%	45%	55%
RS	88%	12%	58%	39%	3%	4%	14%	82%

Abbreviations: *CNB* core needle biopsy, *VAB* vacuum-assisted biopsy, *OE* open excision

**Table 2:** Summary of recommendations of the 3rd Consensus Conference. If a diagnosis was done on CNB, the tailored imaging-guided VAE should be performed with the B3 lesion detection method under consideration of practicability. Only PL without atypia and only PT from the benign and borderline category were considered in the voting

B3 lesions	Diagnosis on CNB	Diagnosis on VAB
ADH	OE	OE. VAE or radiological follow-up justified in selected cases after discussion at MDM
FEA	VAE to complete the removal of the visible lesion	Radiological follow-up justified if the radiological target has been almost completely removed
LN	OE or VAE to complete the removal of the visible lesion	Radiological follow-up if the radiological lesion was removed or OE
PL	OE or VAE to complete the removal of the visible lesion	No further intervention if the radiological lesion was removed completely with VAB
PT	OE (free margins in borderline PT)	OE (if screening-detected) or radiological follow-up (if incidental finding), if the radiological lesion has been removed
RS	OE or VAE to complete the removal of the visible lesion	Radiological follow-up if the radiological lesion has been completely removed

Abbreviations: *CNB* core needle biopsy, *VAB* vacuum-assisted biopsy, *OE* open excision, *MDM* multidisciplinary diagnostic meeting or tumor board

### Classical lobular neoplasia (LN)

#### Histological and radiological features

Classical LN is a neoplastic proliferation of small dyscohesive epithelial cells with origin from the TDLU of the breast. Nowadays, this lesion is classified as a non-obligate precursor of breast cancer, especially of the lobular type [4]. Based on the extent within the TDLUs (<50% and ≥ 50%, respectively) the 2019 WHO classification of Breast Tumors traditionally divides classical LN into atypical lobular hyperplasia (ALH) and lobular carcinoma in situ (LCIS). Since there is no molecular difference between ALH and classical LCIS, and the differentiation of the two lesions suffers from a poor reproducibility between pathologists, lumping these into classical LN is favored [4, 34]. Immunohistochemistry demonstrates the underlying loss of the comprised adherens junction (E-Cadherin, beta-catenin, and p120) for diagnostic reasons. This loss occurs in >80% of cases. Ten to 40% of all LN cases show PIK3CA and CBFB gene mutations [4]. Low-grade DCIS and non-classical LN (such as pleomorphic, apocrine, or florid LCIS) are possible differential diagnoses. They are all classified as B5a lesion and were excluded from this consensus conference since the therapeutic approaches used in these instances vary significantly from those used in B3 classical LN cases [4].

#### Radiological features

Classical LN is an incidental finding in many cases, as it mostly represents a non-palpable, invisible lesion [35, 36]. The lesion is regarded to have no typical imaging pattern, and most calcifications on mammograms that lead to LN diagnosis on VAB occur in co-existing different lesions in the index area, while LN represents a coincidental finding [36]. However, classical LN might be associated with microcalcifications in the mammogram. In rare cases, classical LN presents as a sonographic mass or focal area of subtle non-mass enhancement on MRI [36]. Overall, LN is detected in 0.5–2.9% of CNB and VAB that were performed due to lesions seen on imaging [36, 37].

#### Current evidence for underestimation after CNB/VAB

An upgrade into DCIS or invasive cancer is observed on an average of 20% of cases, with a wide range from 4 to 67% within the current literature [4, 38]. However, if the target imaging lesion is assigned to another histological entity and not to LN following pathological-radiological concordance, the upgrade rate is significantly lower [4, 36, 38–40]. Regarding the upgrade rate, the majority of studies do not differentiate between ALH and classical LCIS. The greatest indicator of an upgrade into invasive cancer, however, is a radiological discrepancy, such as a spiculated mass in clinical imaging and a histopathologic diagnosis of LN in the biopsy specimen of the same lesion [4, 36, 38–41]. After a mammographic-guided VAB with a 7-G needle, the upgrade rate is substantially lower (4%) [38, 40, 42].

In surgical specimens from non-oncologic breast procedures, classical LN frequently represents an incidental finding, for example, in up to 1% in specimens from breast reduction surgery [4]. The relative risk of developing a subsequent invasive breast cancer is 4–10% (1% cumulative risk per year). In most cases, the invasive breast cancer occurs in the same breast (60%), yet the risk is also elevated for the contralateral side (25%) [4, 40]. While positive resection margins with classical LN are of no predictive value, young age and concomitant calcification increase the risk of later cancer [4]. Since LN grows in a dissolute pattern, it is difficult to standardize treatment approaches: hence, debates include OE, life-long follow-up, and chemoprevention in some countries [4].

**Fig. 2:**
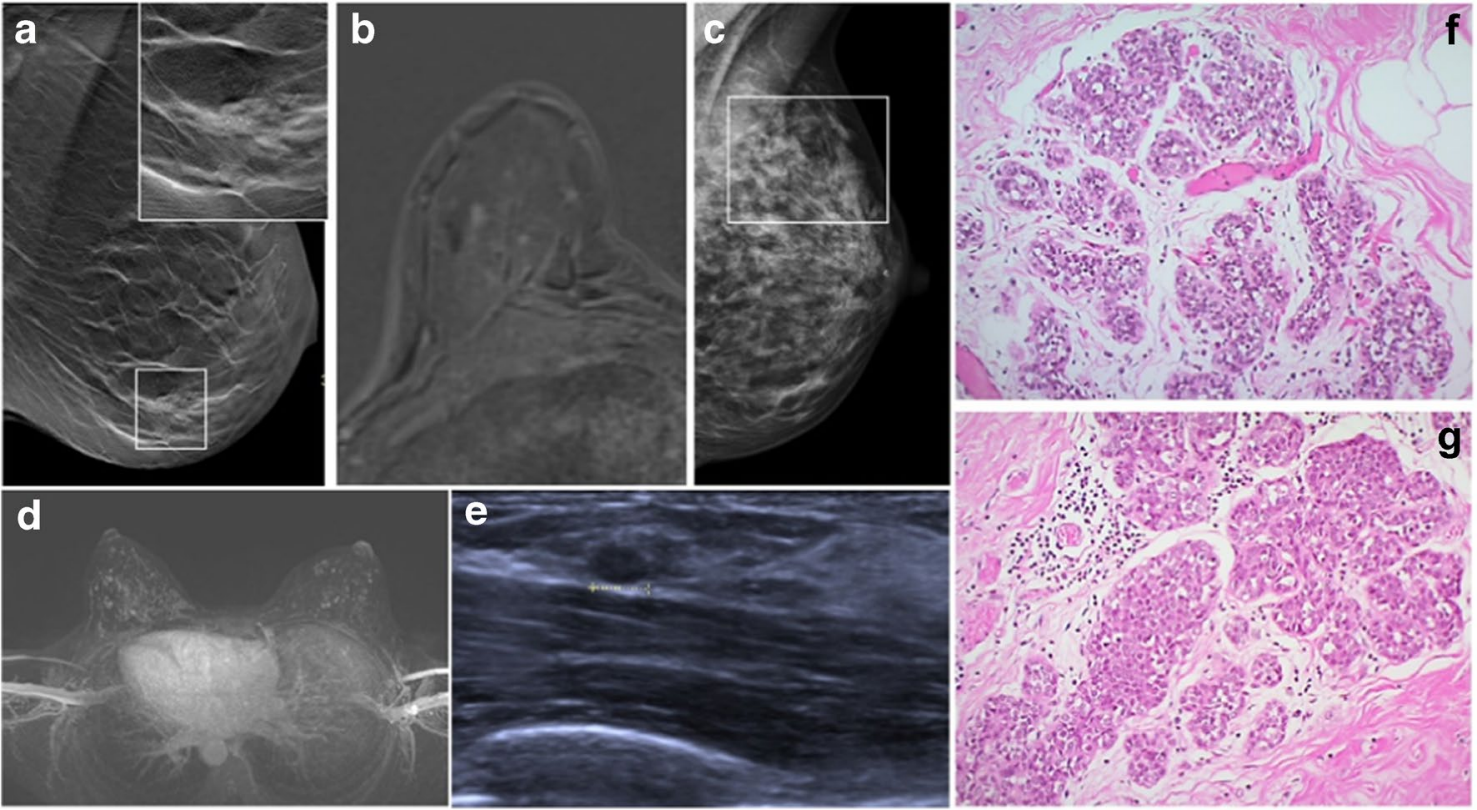
Classical lobular neoplasia (LN). **a** Screen detected calcification (in square) in the breast on mammography. Inset shows clustered calcifications, which were associated to LCIS and adenosis on the subsequent stereotactic vacuum biopsy. **b** Foci corresponding to small areas of LCIS on MRI. **c** Mammography shows dense fibroglandular tissue with diffuse calcifications (in square); the consecutive MRI-guided biopsy confirmed LCIS. **d** Screening MRI shows bilateral strongly enhancing foci within bilateral diffuse non-mass enhancement. **e** The target ultrasound (from the patient in **d**) reveals a small oval mass in the left breast, which was biopsied and histologically confirmed as invasive lobular carcinoma. **d** Morphology of classical LN, type ALH consisting of monotonous cells, subtotally filling the ductular units. **f** Morphology of classical LN, type LCIS, consisting of the same monotonous cells as in **g**, however, almost completely occupying the ductulo-lobular unit

Illustrative morphological and imaging examples of classical LN are shown in Fig. 2.

#### Consensus recommendation of the panel

The panel recommended additional diagnostic/therapeutic procedures following a CNB diagnosis of classical LN with 86% of the votes and a VAE as the next step with 78%. However, the majority of the panelists (90%) would not advise any additional intervention if the radiological target lesion was removed by VAB and instead preferred radiological follow-up (Tables 1 and 2).

### Radial scar (RS)

#### Histological features

Radial scar (RS) or scarring obliterating mastopathy was described in the 1970s [43]. While the term complex sclerosing lesion is often used to describe larger, more disorganized lesions, the terms are frequently used interchangeably. These lesions are characterized by a central fibro-elastotic core with peripherally located compressed glandular structures and cysts, often associated with calcifications and with sclerosing adenosis [4]. In a subset, RS is accompanied by benign epithelial hyperplasia, atypia, or malignant changes, which can be reliably characterized and confirmed by using immunohistochemistry [4, 44]. As described by Rakha et al., the overall upgrade rate in RS with atypia (mostly commonly by ADH or classical LN on CNB) is 25% [44].

#### Radiological features

RS is frequently occult on radiology [45]. If it is evident on clinical imaging, it typically appears on mammography as a stellate lesion with radiolucent center and radiating spicules along with architectural distortion with or without associated calcifications [2, 7, 42]. Tomosynthesis facilitates the recognition of RS on mammogram [46]. On ultrasound, the lesion may appear as parenchymal distortion and/or a hypoechoic mass [47]. On MRI, RS may show a spiculated appearance and architectural distortion with or without enhancement.

These imaging characteristics call for caution since RS may mimic invasive breast cancer [2, 7, 42].

#### Current evidence for underestimation after CNB/VAB

In consecutive OE specimens, the MIBB database revealed a low RS upgrade rate (8%), mostly due to the presence of DCIS [2]. According to the NHS Breast Screening Guidelines, RS upgrade rates were higher if atypia was present (36% vs. <10% without atypia) [7]. A recent Irish study, on a large patient cohort, found similar results, observing an upgrade rate of 9% in RS without atypia, in contrast to an upgrade rate of 33% in RS with atypia [48]. Further studies reported the increased use of therapeutic VAE after CNB diagnosis of RS without atypia with a very low upgrade rate of 0.9–1.6% [46, 49–51]. The number and size of the biopsy specimens have an impact on the histopathological upgrade rate of a targeted OE, similar to other B3 lesions. For example, CNB with 14G needle showed higher upgrade rates than VAB with 8-11G needle (5% versus 1%) [52]. In conclusion, correlation between histology and radiology remains the key feature in the final decision regarding further management.

**Fig. 3:**
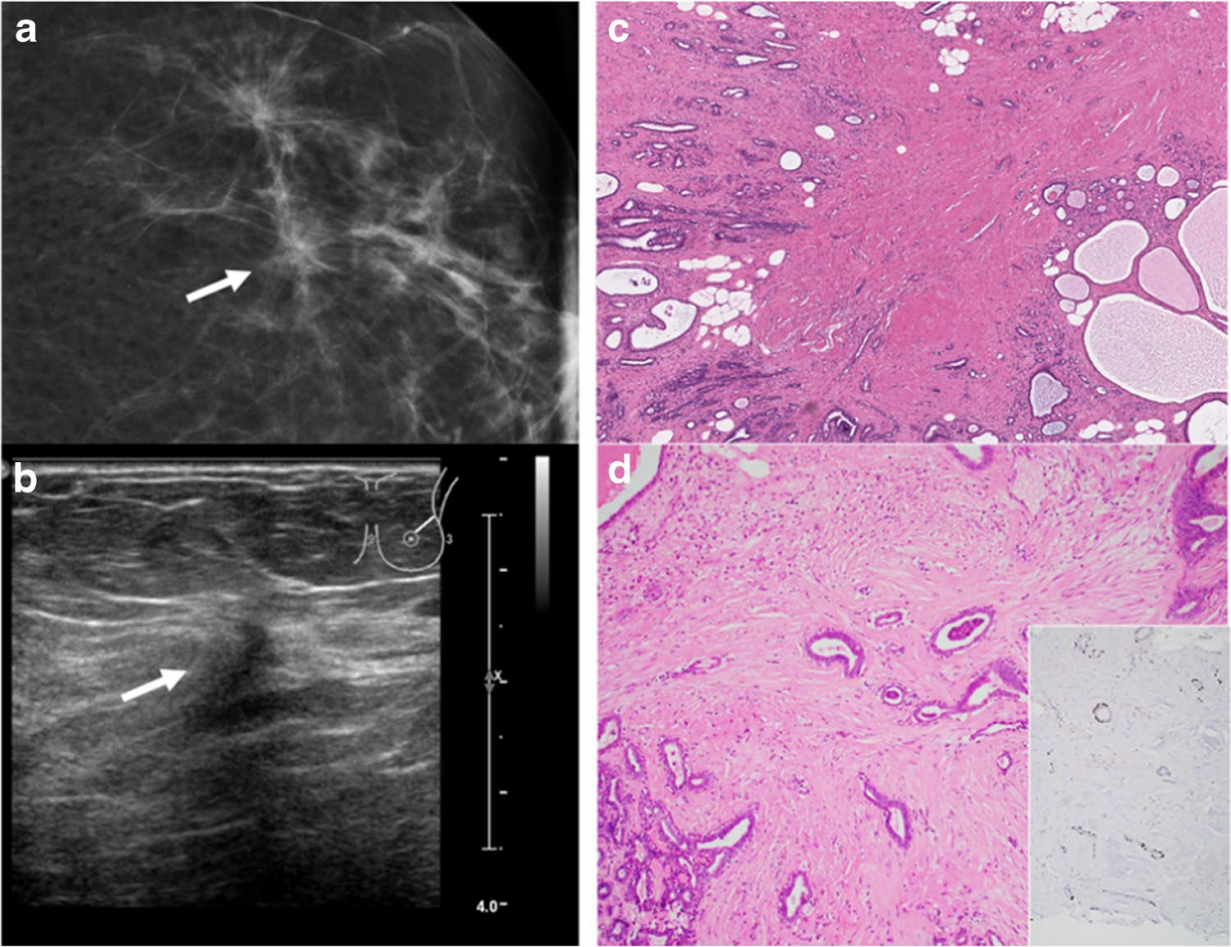
Radial scar/complex sclerosing lesion (RS/CSL). **a** Mammogram demonstrates architectural distortion and asymmetry (arrow). **b** Ultrasound shows an irregular hypoechogenic lesion, corresponding to the mammographic finding (arrow). **c** Histological appearance is characterized by a large central fibroelastotic core with entrapped benign glandular proliferations surrounded by partially cystic benign breast tissue. **d** Benign glandular structures with double layers of ductal epithelial and myoepithelial cells. Inset shows p63 immunohistochemistry highlighting the myoepithelial cells of the entrapped glandular structures

Illustrative morphological and imaging examples of radial scar /complex sclerosing lesions are shown in Fig. 3.

#### Consensus recommendation of the panel

After the identification of RS (without atypia) on CNB in correlation with the imaging size, 58% of the panel supported therapeutic VAE. If the target lesion was entirely eliminated, the majority of the panel (82%) favored radiological follow-up after diagnostic VAB or therapeutic VAE (Tables 1 and 2).

### Papillary lesions (PL)

#### Histological features

PL represent a large spectrum of histopathological entities including benign pure papillomas, papillomas with concomitant atypical lesions (ADH/LN/DCIS), papillary DCIS, encapsulated and solid papillary carcinomas [4]. The corresponding B-classification varies from B3, B4, to B5a or even B5c [1]. Some guidelines advocate classifying a lesion as B2 rather than B3 if a small intraductal papilloma (<1mm) has been completely removed in the CNB/VAB; however, this is not uniformly accepted [1]. ADH identified within an intraductal papilloma may have a maximum dimension of 3 mm as described by the WHO classification, as opposed to a pure ADH (whose maximum diameter remains 2 mm) [4]. In this conference, only pure papilloma forms without atypia was taken into account in the voting and referred to as PL, but PL with atypia was discussed. Pure PL are situated in dilated ductal spaces and are composed of an arborising fibrovascular core and a corresponding heterogeneous (molecularly polyclonal) ductal epithelial/myoepithelial proliferation analogous to usual ductal hyperplasia (UDH) [4]. Additional immunohistochemical stains (such as ER, CK5, and p63 or other myoepithelial markers) can aid to distinguish the lesion and determine whether the biopsy contains a pure papilloma, an atypical papilloma, or a papillary DCIS when there is any diagnostic uncertainty [4]. The presence of mosaic type basal keratin (e.g., CK5) stain and a heterogeneous ER expression reliably confirms a pure papilloma with usual epithelial proliferation, although PL without atypia may also highly express ER. In contrast, loss of CK5 and uniform ER expression are typically seen in atypical papillary lesions [4].

#### Radiological features

On mammography and ultrasonography, intraductal PL typically appear as hypoechogenic, circumscribed lesions, sometimes with peripheral vascularization. Occasionally, they show architectural distortion [42, 51, 53–55]. Linear calcifications organized as a branching bush can be encountered as a typical appearance. Pleomorphic calcifications can occasionally be seen within a circumscribed mass [42, 51, 53–55]. PL typically appear as circumscribed, solid enhancing lesions on MRI; however, they can also show irregular shapes and ill-defined margins [56].

#### Current evidence for underestimation after CNB/VAB

Although PL with or without atypia (such as ADH or classical LN) are both categorized as B3 lesions, the management and biological behavior in both instances are entirely different [1, 2, 4]. Pure intraductal PL have a remarkably low upgrade rate (1–9%), whereas lesions with concomitant atypia have been reported to have a higher upgrade rate of up to 38% [2, 4]. There are numerous predictors of prognosis for these two biologically distinct lesions, including size >1cm, symptomatic lesions, peripheral location >5cm distance from the nipple, concomitant calcifications, and multiple lesions [2, 53, 55, 57–59]. Since the last consensus conference in 2018, 47 studies that distinguish between benign pure PL and PL with atypia have been published, most of which provided upgrade rates. When assessing the results, the majority of them gathered data from a median of 139 lesions per study without atypia and a median of 97 lesions per study with atypia. Most studies employed CNB for biopsy. The median upgrade rate for PL without atypia to DCIS or invasive carcinoma, according to these most recent pooled analyses, was only 2.3 % [2, 53, 55, 57–59]. In contrast, PL with atypia (at least ADH, LN) had a significantly higher upgrade to DCIS or invasive carcinoma (median 26.9%). The risk of subsequent breast cancer is two times higher in patients with PL without atypia and 5 to 7 times higher in patients with PL with atypia compared to the normal population [53–55, 57–59].

**Fig. 4:**
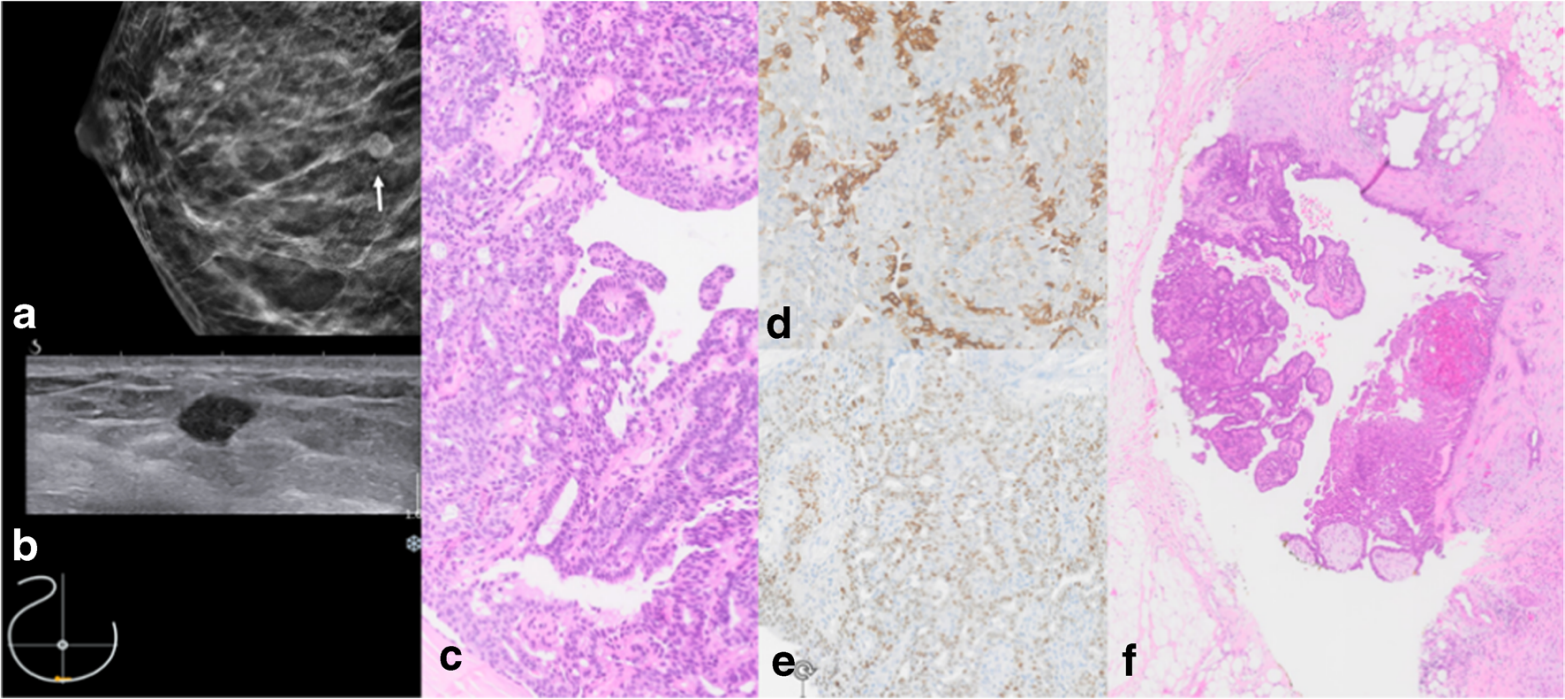
Papilloma without atypia. **a** Right cranio-caudal (cc) mammography image with hyperdense circumscribed mass lesion (white arrow). **b** Correlating small hypoechoic mass on ultrasound. **c** H&E stain of the core needle specimen shows papilla with a fibrous stroma and a heterogeneous mixture of cytologically bland ductal epithelium and myoepithelium. **d** CK5/6 mosaic pattern and **e** heterogeneous mostly weak to moderate ER expression. **f** Open excision (OE) specimen confirms benign papilloma

Illustrative morphological and imaging examples of papillary lesions are shown in Fig. 4.

#### Consensus recommendation of the panel

The panel almost equally suggested OE or therapeutic VAE following a CNB-based diagnosis of PL without atypia (45% vs 55%). If the target lesion was completely removed, the whole panel (100%) preferred radiological follow-up after diagnostic VAB (Tables 1 and 2).

### Flat epithelial atypia (FEA)

#### Histological features

**Fig. 5:**
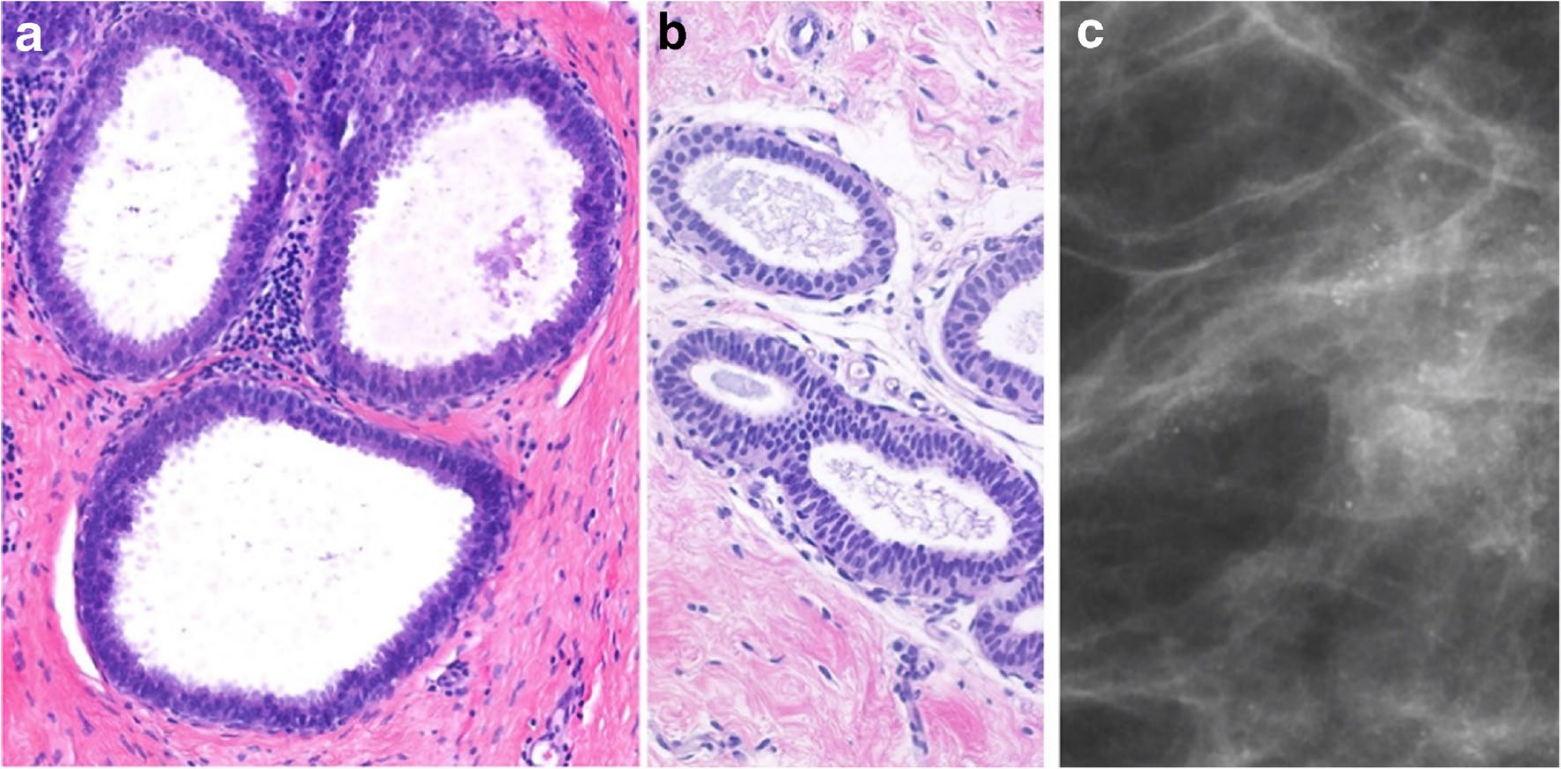
Flat epithelia atypia (FEA). **a** One to few layers of cells with low grade atypia covering a dilated acinus. **b** Ductuli covered by pseudostratified columnar epithelium with low grade atypia (H&E stain). **c** Radiological illustration shows regional amorphous microcalcifications, not confirming a duct distribution associated to FEA (mammography image, magnification view)

FEA belongs to the columnar cell lesions (CCL) of the breast and is characterized by one to several layers of mildly atypical cuboidal to columnar cells resembling the monomorphic cytological atypia of low-grade DCIS (Fig. 5) [4]. The current WHO classification distinguishes between columnar cell lesions, which per definition lack nuclear cytological atypia and are designated a B2 lesion, and FEA, which exhibits a flat lesion with nuclear atypia and represents as B3 lesion [2, 4]. Since FEA shares molecular changes of the so-called low-grade molecular pathway with other low-grade lesions like ADH, low-grade DCIS, or classical LN, and even with tubular carcinomas, it is frequently associated with these other lesions [4]. FEA often occurs in dilated TDLU with associated secretions and calcifications. The distinction between FEA and CCL without atypia is performed only on H&E histology since both lesions share the same immunoreactive profile showing strong ER positivity, CK5 negativity, and a low Ki-67 labeling index (8% on average) [2, 4].

#### Radiological features

Isolated FEA is rarely detected; instead, it is typically seen in combination with other suspicious lesions and shares imaging characteristics with both malignant and benign lesions [60, 61]. Image features may thus be commonly related to concurrent other pathology. On mammography, FEA-associated calcifications are mostly amorphous or fine pleomorphic and clustered. On ultrasound, FEA may appear as rather irregular, microlobulated, or hypoechoic mass [60, 61]. On MRI, FEA may be occult or appear as a mass with non-specific features or as non-mass enhancement [62].

#### Current evidence for underestimation after CNB/VAB

The evidence on the biological behavior of FEA is limited. Although some cases of FEA may progress to invasive carcinoma, the risk of progression appears to be very low compared to the risk associated with ADH and LN. In addition, the risk of FEA may be determined by potential concomitant ADH and LN [2, 4]. Although a few studies have shown that CCL without atypia are associated with a slightly increased (1.5-fold) risk for subsequent development of invasive breast cancer, this risk is not clearly independent of the risk associated with concomitant proliferative lesions [4]. Up to 30% malignancy (DCIS or invasive breast cancer) may appear in the subsequent VAE or OE after FEA on CNB [1, 2, 4]. The upgrade rate of following OE was examined in several major review articles with somewhat different estimates ranging from 1 to 8% [50, 63, 64]. However, if more than 90% of the targeted calcifications have been eliminated, recent data, including individual trials, support the idea of therapeutic VAE with radiological follow-up [50, 63, 64]. In the MIBB database, the upgrade rate was rather high (16%, with half to invasive carcinoma or DCIS) [2]. Several independent groups and international guidelines suggest a case-by-case discussion or radiological follow-up as the preferred course of action if FEA is diagnosed on VAB. Only instances with pathological-radiological discordance, mass lesions, or cases with residual calcifications after biopsy should be treated with OE [1, 2, 65–70]. Age, imaging presentation, other breast cancer risk factors, the size of lesions, and associations with calcification in addition to radiological-pathological correlation of FEA are key aspects for informed decision-making [21].

#### Consensus recommendation of the panel

Depending on the clinical presentation and the size of the lesion in the clinical imaging, the panel preferred either VAE or OE if FEA is identified on CNB. The majority of panelists are confident in surveillance and radiological follow-up if FEA is returned on VAB and >90% of the target lesion, such as microcalcifications, has been eliminated (Tables 1 and 2).

### Benign and borderline phyllodes tumors (PT)

#### Histological features

PT are very rare circumscribed fibroepithelial lesions characterized by an exaggerated intracanalicular growth of a molecularly clonal hypercellular stroma resulting in leaf-like fronds covered by benign epithelium. Less frequent, a pericanalicular growth pattern with concentrically arranged stroma around ducts may occur [4]. PT are considered de novo lesions, but there is some evidence that a small subset may develop from fibroadenoma. On the molecular level, MED12 or TERT promoter mutations are most frequent but also other mutations have been identified in PT (e.g., ENT, EGFR, c-KIT, NF1, PTEN, p53). PT are associated with Li-Fraumeni syndrome. Based on distinct characteristics such as mitotic activity, stromal cellularity, borders, and the ratio of epithelial to stromal components, the WHO 2019 edition divides PT into benign, borderline, and malignant categories [4]. While malignant PT is classified as B5 lesion and has not been taken into consideration for this conference, benign and borderline PT are considered B3 lesions and were discussed at this meeting [1, 2]. The interobserver agreement between pathologists in the differential diagnosis between cellular FA and benign PT seems to be problematic, particularly on biopsies, but their distinction from borderline and malignant PT seems to be considerable [71–74]. To avoid overtreatment, the WHO classification advises classifying the lesion as FA in cases of histological ambiguity between FA and PT [4]. European guidelines, however, endorse the use of the term “benign fibroepithelial tumor, B3” in needle biopsies if no definitive diagnosis can be made. The majority of PT is benign, whereas borderline and malignant PT occur less frequently [75].

#### Radiological features

On imaging, PT can appear as a progressively growing fibroadenoma. On sonography, PT typically appears as a well-defined mass with heterogeneous echogenicity and may be oval or lobulated with cystic spaces. On mammography, PT appears as a well-defined mass without calcifications [76]. On MRI, benign PT may resemble FA, although it typically has a more irregular shape, ill-defined margins, and echogenic heterogeneity. MRI characteristics of benign PT can overlap with those of malignant PT and are of low predictive value [77]. Malignant PT usually shows imaging characteristics that are typical for malignancy, such as irregular shape and contrast medium wash out phenomenon (kinetic curve type III) [78–80].

#### Current evidence for underestimation after CNB/VAB

After CNB or VAB, the upgrade to malignancy is uncommon. However, the definitive histological diagnosis can best be rendered on OE specimens where completeness of the excision can be evaluated [2]. Clinical and radiological features should be discussed preoperatively with the pathologists in a multidisciplinary board. The distinction between cellular FA and benign PT may present a diagnostic challenge for pathologists and radiologists [78–83].

**Fig. 6:**
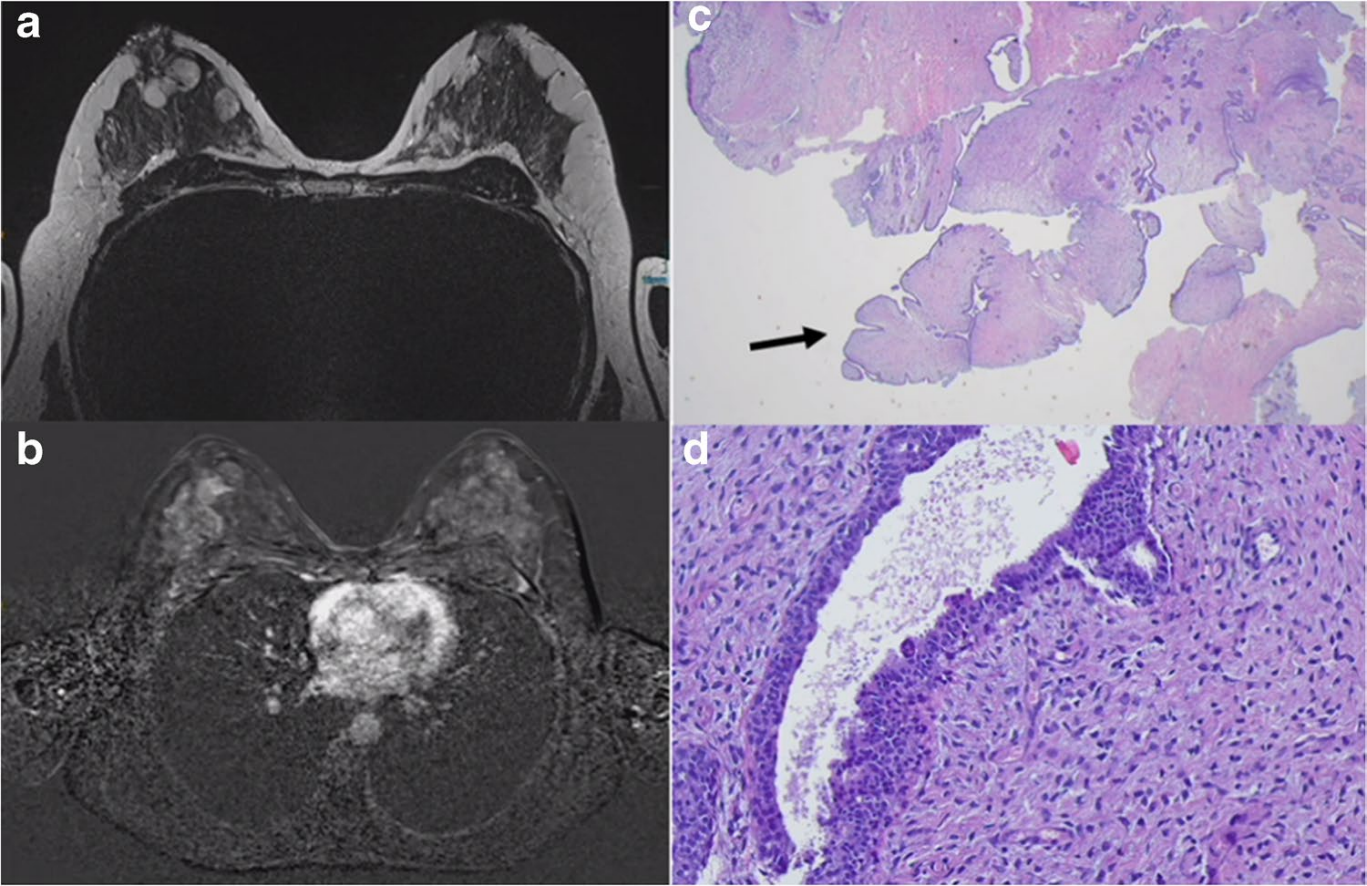
Phyllodes tumor (PT). **a** and **b** Magnetic resonance imaging (MRI) of a large benign PT in the breast. **c** Core needle biopsy reveals a fibroepithelial tumor with leaf-like structures (arrows) and **d** hypercellular stroma without atypia (H&E stain)

Illustrative morphological and imaging examples of PT are shown in Fig. 6.

#### Consensus recommendation of the panel

**Fig. 7:**
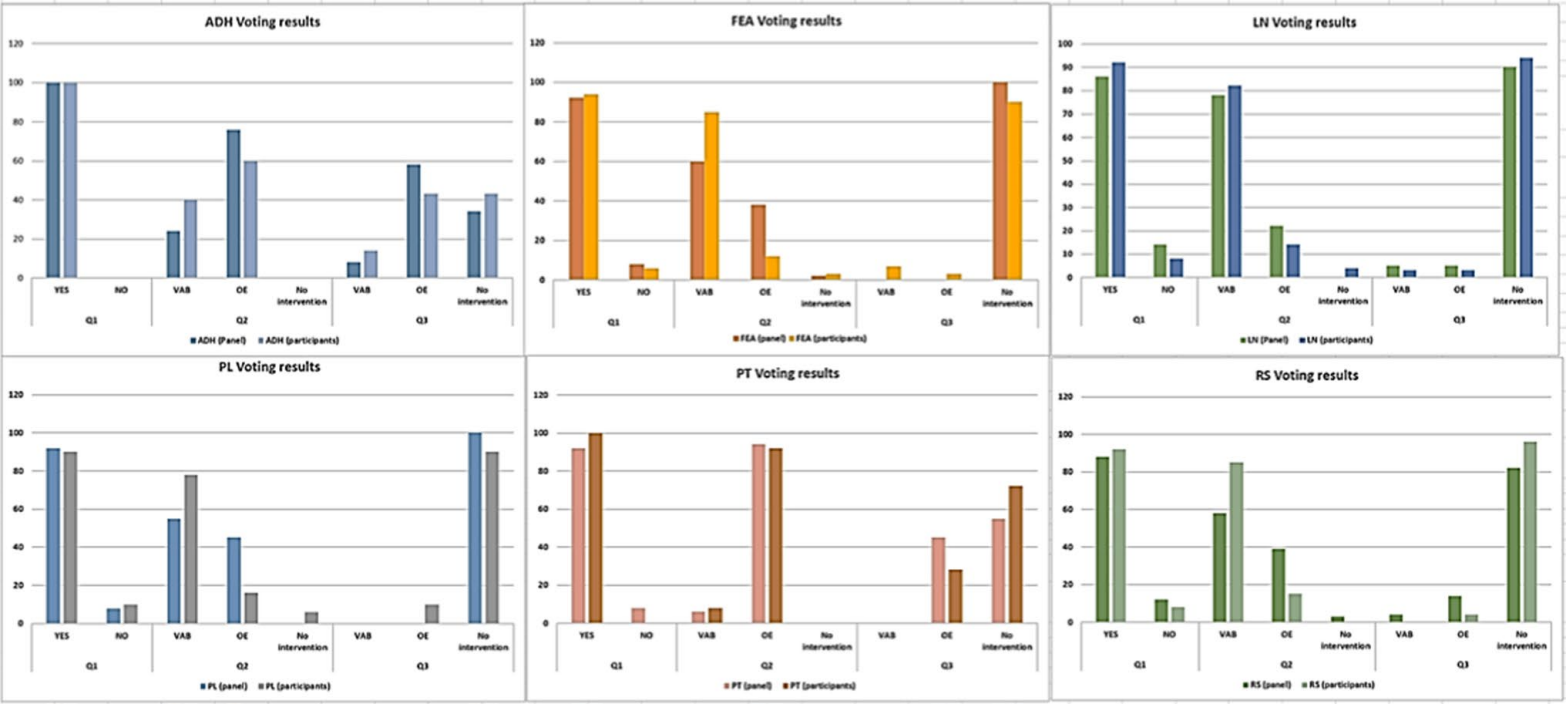
Results of vote by the panel and conference participants. Participants and panelists favored a relatively similar clinical approach for all B3 lesions; differences in the subsequent steps (diagnostic or surgical) were statistically negligible (chi-square test)

The majority of the panel recommended OE after CNB diagnosis of PT (92%). If a B3 PT diagnosis is made on VAB, the option of OE or follow-up with no further intervention are both justified if the lesion is radiologically removed (Tables 1 and 2, Fig. 7).

## Discussion

Since the 2nd Consensus Conference in 2018, multiple further studies on B3 lesions have been published, and, therefore, the interdisciplinary and international consensus recommendations needed to be updated. The 3rd Consensus Conference followed the pre-defined upper and lower risk rates for B3 lesions, defining that the underestimation rates should be below 5% for invasive carcinomas and below 10% for DCIS [2]. This year, the panel added diagnostic VAB, therapeutic VAE, OE, or no further intervention with radiological follow-up to the voting options. The panel examined the option for a second vacuum assisted biopsy in the sense of a therapeutic VAE in certain situations for lesions with an upgrade rate of >10%, which mostly affects ADH and classic LN.

If the CNB resulted in a B3 lesion, removal of the lesion was recommended by the panelists; in cases of ADH 100% (2018: 100%), FEA 92% (2018: 65%), LN 86% (2018: 67%), PL 92% (2018: 77%), PT 92% (2018: 98%), and RS 88% (2018: 60%) [9]. These slightly increased numbers imply that the more established option of VAE is used as an alternative to OE in routine clinical practice.

In cases with ADH, there was no clear preference for a de-escalation strategy despite it being VAB detected. The majority of the panelists recommended a further OE which was similar to 2018 but on an upward trend (58% versus 51%) [9]. Similar to the 2018 advice, when 12% recommended OE in cases of LN diagnosed using VAB, a de-escalating strategy with merely radiological follow-up was preferred by 90% of panelists in instances with visible lesions in classical LN. This is true despite the variable risk for an upgrade which can be as high as 20% in the presence of certain risk factors. Current data imply a more individualized decision, e.g., under consideration of the number of LN foci in the biopsy specimen [38]. We found no significant difference in the diagnostic and therapeutic recommendation from the panelists if the B3 lesion was completely removed by VAB on clinical imaging; in FEA (100% voted for no further intervention compared to 97% in 2018), PL (100% no further intervention compared to 98% in 2018), and RS (82% no further intervention versus 89% in 2018).

In the diagnosis of PT on VAB, there was a clear trend towards OE (45%) compared to the recommendations in 2018 (OE 8%; no further intervention 88%).

A large UK series with more than two million screening CNB yielded that B3 lesions without histological atypia have lower upgrade in the subsequent VAB or OE. However, there was a trend for VAE in the majority of patients with B3 lesions, independent of the presence of atypia [42]. The VAB needle gauge size has increasingly been a subject of discussion as more tissue volume can yield a higher positive predictive value (“size matters”), as was demonstrated in a large study including >6300 pooled patients from 16 countries, putting the diagnostic VAB equivalent to VAE [50]. Larger VAB specimens (7-8 G) with more than 12 tissue samples have an approximate weight of 4 g and thus have a comparable volume /weight to a therapeutic OE of B3 lesions [7]. Especially in RS, FEA, PT, and PL without atypia, the current literature supports the option of increased use of therapeutic VAE and active surveillance when the lesion is completely removed in the clinical imaging. However, one needs to keep in mind that financial reimbursement of diagnostic VAB and therapeutic VAE is different from country to country and in some countries therapeutic VAE is yet to be financial compensated. This may result in different logistic possibilities, but our consensus report may lead to a higher acceptance and wider implementation of therapeutic VAE. The recommendation for excision of most B3 lesions with VAE or OE is reliant on the approach. In the present discussion, an upper risk limit of 5% upgrade for invasive carcinoma and 10% upgrade for ductal carcinoma in situ (DCIS) was agreed on. Following a CNB diagnosis for B3 lesions in countries without any possibility of removal by VAE, an OE should be considered. In selective cases, depending on the individual patient characteristics (e.g., age, family history), a de-escalation strategy with surveillance can be considered, especially for lesions with low upgrade rates.

A complete radiological removal of the lesion is considered to be a prerequisite for avoiding further interventions.

Above all, it should be emphasized that the decision on further management of B3 lesions needs to be based on a careful discussion in a multidisciplinary conference in order to correlate the histological and radiological findings.

The statements of the 3rd International Consensus Conference have some limitations. The B-categorization of breast lesions is not established worldwide. Furthermore, the VAB-technique of breast lesions is not yet established in all countries, and to replace open surgical intervention by VAB, the operator must be trained in this technique prior to applying. The 2019 WHO classification endorses the term papilloma with ADH/DCIS (instead of the less precise term of atypical papilloma). We used this terminology “papilloma with concomitant atypical features (such as ADH/LN/DCIS)” in the general description of papillary lesions. The voting only focused only on pure papillomas, whereas papillomas with concomitant atypical features (such as ADH/LN/DCIS) were excluded from the voting. The Consensus Conference did not consider B3 lesions without radiological presentation. These B3 lesions, which are accidental findings in CNB for associated lesions, present a challenge to clinicians. Radiological surveillance is not reliable in these cases; furthermore, targeted open surgical resection or VAB cannot be performed. Likewise, other consensus conferences such as the St. Gallen Breast Cancer Conference [84] and the Consensus Conference on B3 lesions reflect expert opinions and recommendations based on current studies and literature. The results of both previous Consensus Conferences on B3 lesions have been considered in several international guidelines [85]. It was not our intention to replace the guideline-building process by the voting process. The specific and unique follow-up plan for each lesion was not included in the panel’s debate. Nevertheless, depending on the initial lesion-detecting technique, the patient’s age, clinical variables, and other considerations, at least an annual follow-up with radiological control was recommended. Annual mammography should be advised for patients with LN or ADH and a known higher risk of breast cancer.

## Data Availability

The datasets used and analyzed during this study are available from the corresponding author on reasonable request.
